# Dynamic of bacterial and archaeal diversity in a tropical soil over 6 years of repeated organic and inorganic fertilization

**DOI:** 10.3389/fmicb.2022.943314

**Published:** 2022-08-16

**Authors:** Sophie Sadet-Bourgeteau, Christophe Djemiel, Nicolas Chemidlin Prévost-Bouré, Frederic Feder

**Affiliations:** ^1^Agroécologie, INRAE, Institut Agro Dijon, Université Bourgogne, Université Bourgogne Franche-Comté, Dijon, France; ^2^CIRAD, UPR Recyclage et Risque, Montpellier, France; ^3^Recyclage et risque, Univ Montpellier, CIRAD, Montpellier, France

**Keywords:** organic and inorganic fertilization, high-throughput sequencing, tropical, soil, microbial communities

## Abstract

The soil microbial community plays important roles in nutrient cycling, plant pathogen suppression, decomposition of residues and degradation of pollutants; as such, it is often regarded as a good indicator of soil quality. Repeated applications of mixed organic and inorganic materials in agriculture improve the soil microbial quality and in turn crop productivity. The soil microbial quality following several years of repeated fertilizer inputs has received considerable attention, but the dynamic of this community over time has never been assessed. We used high-throughput sequencing targeting 16S ribosomal RNA genes to investigate the evolution of the bacterial and archaeal community throughout 6 years of repeated organic and inorganic fertilizer applications. Soils were sampled from a field experiment in La Mare (Reunion Island, France), where different mixed organic-inorganic fertilizer inputs characterized by more or less stable organic matter were applied regularly for 6 years. Soil samples were taken each year, more than 6 months after the latest fertilizer application. The soil molecular biomass significantly increased in some organically fertilized plots (by 35–45% on average), 3–5 years after the first fertilizers application. The significant variations in soil molecular microbial biomass were explained by the fertilization practices (cumulated organic carbon inputs) and sometimes by the soil parameters (sand and soil carbon contents). The structure of the bacterial and archaeal community was more influenced by time than by the fertilization type. However, repeated fertilizer applications over time tended to modify the abundance of the bacterial phyla *Acidobacteria, Actinobacteria, Bacteroidetes, Firmicutes*, and *Proteobacteria.* To conclude, the present study highlights that the soil bacterial and archaeal community is lastingly modified after 6 years of repeated fertilizer inputs. These changes depend on the nature of the organic input and on the fertilization practice (frequency and applied quantity).

## Introduction

To meet the global demand for food production, fertilization is a common/basic farming practice usually repeated every year to maintain soil fertility and crop yields. In parallel, intensive agricultural practices contribute to decrease the soil organic matter content, with negative consequences on soil fertility (Clapp et al., 1986; Tate, 1987). One way of reversing this degradation is to increase the soil organic matter content by applying organic fertilizers such as manures, composts, or crop residues (Hutchinson et al., 2007; Peltre et al., 2012). In the field, organic fertilizers are often used in association with inorganic fertilizers to improve crop productivity. However, assessing the effect of these practices on soil quality is important to reach a sustainable management of agriculture. Soil quality was defined as “the capacity of a soil to function within ecosystem and land-use boundaries to sustain biological productivity, maintain environmental quality, and promote plant and animal health” by Doran and Parkin (1994, 1997). As underlined by Bünemann et al. (2018), biochemical and biological indicators are potentially highly valuable tools for assessing soil quality. The most frequently used biochemical indicators are soil organic matter/carbon and the pH (Bünemann et al., 2018). As for soil biological indicators, microorganisms are essential for soil quality because they play a key role in soil functioning (Vivant et al., 2013; Tardy et al., 2014; Maron et al., 2018; Abis et al., 2020). The most frequently used microbiological indicator is microbial biomass (Bünemann et al., 2018). However, recent advances in soil biology—more precisely molecular methods based on DNA—provide access to and describe a larger part of the soil microbiota. Thus, other indicators focused on the soil microbial diversity—e.g., taxonomic richness or community composition—are now used to assess the effect of agricultural practices on soil quality (Tardy et al., 2015; Chemidlin Prévost-Bouré et al., 2021; Dunn et al., 2021).

Several studies have compared the impact of repeated organic or inorganic fertilization on soil biochemical and biological indicators. There is no consensus in the literature about soil biochemical indicators. Some studies highlight no difference in the pH of unfertilized and organically/inorganically fertilized soils (Gu et al., 2009; Ho et al., 2015; Chen et al., 2016; Li et al., 2017; van der Bom et al., 2018; Guo et al., 2019; Diallo et al., 2021). Other works show an impact of fertilization practices: sometimes the soil pH decreases whatever the fertilization type (Li et al., 2015; Sun et al., 2015; Francioli et al., 2016), and sometimes it increases under organic or combined inorganic-organic fertilization (Zhong et al., 2010; Dong et al., 2014; Hartmann et al., 2015; Ding et al., 2016; Zhao et al., 2016; Wang et al., 2017; Feder, 2021a,b). As regards the soil organic carbon content, an increase is overall observed whatever the fertilization type considered (organic or inorganic, or a combination of both) (Hao et al., 2008; Zhong et al., 2010; Chakraborty et al., 2011; Dong et al., 2014; Zhang et al., 2014; Hartmann et al., 2015; Li et al., 2015, 2017; Sun et al., 2015; Francioli et al., 2016; Kumar et al., 2017; Wang et al., 2017; Guo et al., 2019). However, some of these studies also highlight a greatest increase of the soil organic carbon content when organic material is used (alone or combined with an inorganic material) compared with inorganic fertilization (Hao et al., 2008; Zhang et al., 2014; Hartmann et al., 2015; Li et al., 2015; Sun et al., 2015; Francioli et al., 2016; Wang et al., 2017; Guo et al., 2019). This suggests that fertilization practices including organic material are a better way to improve soil fertility, organic matter content (Mäder et al., 2002; Liang et al., 2012), and consequently to enhance soil microbial community (Esperschütz et al., 2007; Gomiero et al., 2011).

The literature is more consensual about soil biological indicators. Most studies show an increase of the soil microbial biomass after repeated organic or inorganic fertilization, or a combination of the two fertilization types (Stark et al., 2007; Hao et al., 2008; Gu et al., 2009; Liu et al., 2009; Chakraborty et al., 2011; Zhang et al., 2012; Li et al., 2015; Francioli et al., 2016; Kumar et al., 2017; Guo et al., 2019). The soil microbial biomass increases more when organic material is used in addition to inorganic fertilizer (Hao et al., 2008; Chakraborty et al., 2011; Zhang et al., 2012; Francioli et al., 2016; Kumar et al., 2017; Guo et al., 2019). Most studies also highlight that organic inputs (alone or combined with mineral fertilizers) increase the soil microbial diversity compared with sole mineral fertilizer applications (Zhong et al., 2010; Kamaa et al., 2011; Zhang et al., 2012; Chen et al., 2016; Francioli et al., 2016). Organic fertilization also modifies the soil microbial community composition by stimulating microbial groups (*Firmicutes* and *Proteobacteria*) known to prefer nutrient-rich environments and involved in the degradation of complex organic compounds (Hartmann et al., 2015; Francioli et al., 2016; Wang et al., 2017; van der Bom et al., 2018). Interestingly, among the soil microbes involved in soil functioning, bacteria appear more sensitive than fungi to fertilization practices (Ai et al., 2018). This suggests that the bacterial community is a good indicator to assess the effect of these practices on the soil (Kamaa et al., 2011; Chen et al., 2016; Francioli et al., 2016).

The different literature results about soil biochemical indicators could be explained by different experimental conditions, i.e., the duration of repeated fertilization, the rate of fertilizer applications, the time lapse between fertilizer application and soil sampling, the quality of the different organic inputs, and the soil type. Nevertheless, despite these differences, one consensual observation is that repeated application of organic material mixed with inorganic material improves the soil microbial quality by increasing soil microbial biomass and diversity. To our knowledge, the majority of studies assessed soil quality following several years of repeated fertilizer inputs, but did not address the dynamic of the soil community over the years. The dynamic of the soil microbial community has been assessed over several months after a single organic fertilizer application (Calbrix et al., 2007; Bastida et al., 2008). These works reveal that organic fertilization initially induced strong changes in bacterial functional and genetic structures during the first 3 months after organic fertilization, followed by a period of resilience leading to similar communities in both the fertilized and control plots after 6 months (Calbrix et al., 2007). However, this transient effect of a single dose of organic fertilizer (3–6 Mg ha^–1^; Calbrix et al. (2007)) on the soil microbial community seems dependent on the applied quantity; since a persistent effect has been observed with 195 Mg ha^–1^ of organic material (Bastida et al., 2013). Therefore, determining when the soil microbial communities are persistently modified in response to repeated fertilizer inputs (type and quantity) is important to improve fertilization practices.

The present study takes advantage of a mid-term field experiment in La Mare, Reunion Island (France). The site was laid out for the experiment in 2013, and different mixed organic/inorganic fertilizer inputs were applied from March 2014. Soil samples were generally collected 1 year after the organic-inorganic material application every year for 6 years to monitor microbial community changes. The experiment involved different types of organic inputs and fertilization practices. The responses of the bacterial and archaeal community to organic-inorganic inputs were assessed by high-throughput sequencing targeting 16S ribosomal genes. Based on previous works, we hypothesized that the soil microbial community indicators (microbial biomass, diversity and composition) would not increase or change in the first years of fertilizer inputs, and that lasting modifications would occur several years after repeated fertilizer inputs. The time needed for this modification to occur is not documented in currently available literature. We also hypothesized that this interval would be dependent on the fertilization practice (frequency of application, quality and quantity of organic material inputs).

## Materials and methods

### Experimental sites, soil sampling strategy, and soil chemical analysis

The experiment was conducted at the SOERE PRO^1^ experimental station of La Mare (Sainte-Marie), Reunion Island (20°54’12.2”S 55°31’46.6”E). The soil is a Nitisol (Feder, 2013; WRB, 2015) containing 42% clay, 47% silt, and 11% sand on average in its tilled layer. The climate is tropical with a mean rainfall of 2,000 mm year^–1^ and a mean annual temperature of 25°C. The trial was planted in March 2014 from viable buds of the R579 sugarcane variety with 1.5 m spacing between rows. Four different organic materials representing the typical organic fertilizers found in Reunion Island were applied: (1) liquid pig manure (LP); (2) poultry litter (PL); and (3) two similar sewage sludge materials (SS1 and SS2). These organic treatments were compared with a control treatment that did not receive any organic input (CON). Each treatment was replicated three times within a randomized complete block design. Each plot was composed of 6 sugarcane rows 28 m in length, making up a total plot area of 250 m^2^. Additionally, all plots received mineral fertilization adjusted to annual sugarcane needs (Supplementary Table 1). SS1 and LP were applied every year, in February and November 2014, November 2015 and 2016, October 2017, 2018, and 2019 (Supplementary Table 1). SS2 and PL were applied every 3 years, in February 2014 and October 2017 (Supplementary Table 1). The amounts of organic inputs are given in Supplementary Table 1. The nature and doses of organic fertilizers and the associated mineral supplementation are representative of the local context in Reunion Island. The requirements for sugar cane to reach a yield of ca. 120 T/ha are 180, 60, and 230 kg/ha of N, P, and K, respectively; these values are similar to recommendations in other situations (Leal et al., 2009; Blum et al., 2013; Ghube et al., 2017). The sugarcane yields are given in Supplementary Table 2. Each plot was equipped with a TDR CS 650 probe to measure the moisture level every hour at the following depths: 10, 30, 50, 70, 90, and 110 cm. Irrigation was adjusted according to rainfall so that the soil never underwent an intense or long dry period. A description of the pesticides treatments is given in Supplementary Table 3. We conducted the initial soil characterization of the site in November 2013, after plowing the plot. In March 2014, sugarcane cuttings were buried and there was no further tillage thereafter. The following years, soils samples were collected just after the sugar cane harvest and before the organic fertilizer applications in November 2013, 2014, 2015, and 2016, and in October 2017, 2018, and 2019 on bulk soils. In each plot, 5 soil cores (Ø 7 cm) were randomly taken at 0–25 cm depth, mixed and homogenized by 4 mm mesh sieving, so that above-ground plant debris, roots and stones were also removed. This led to a total of 105 soil samples (5 treatments × 3 replicates × 7 years). A portion of each soil was air-dried for physico-chemical analysis: particle size distribution, pH, soil organic carbon, soil total nitrogen (N), soil C/N ratio, total phosphorus (P), and Cation Exchange Capacity (CEC). These analyses were performed at the COFRAC-accredited INRAE Soil Analysis Laboratory (ARRAS, France).^2^ The mean soil physico-chemical data measured between 2013 and 2018 are given in Table 1. The rest of the sieved soil was lyophilized and stored at −40°C prior to DNA extraction and molecular analyses.

**TABLE 1:** Physico-chemical parameters of soils from La Mare (Reunion Island) according to the fertilizer input (CON, inorganic fertilized control; SS1 and SS2, sewage sludge inputs; LP, liquid pig manure; PL, poultry litter) and the sampling time.

Treatment	Organic carbon	Total N	P O-Dabin	C:N	pH (water)	CEC
	g kg^–1^ DM					Cmol_(+)_ kg^–1^ DM
**CON**						
2013	19.75	1.73	0.15a	11.42ab	6.1	10.79
2014	20.21	1.80	0.13ab	11.25b	6.06	10.91
2015	19.82	1.75	0.15a	11.31b	6.19	11.02
2016	20.58	1.80	0.13ab	11.4ab	6.25	11.95
2017	20.29	1.70	0.13ab	11.89ab	6.22	12.97
2018	20.79	1.76	0.1b	11.78ab	6.37	13.29
2019	20.83	1.71	0.12ab	12.18a	6.3	13.02
Effect of sampling time^†^	NS	NS	**	*	NS	NS
**SS1**						
2013	21.19	1.78	0.10	11.86	6.01b	10.52c
2014	20.86	1.84	0.11	11.35	5.98b	10.50c
2015	20.06	1.78	0.12	11.25	6.02b	11.02bc
2016	21.50	1.9a	0.11	11.31	6.25ab	12.55ab
2017	21.40	1.79	0.10	11.98	6.16ab	13.00a
2018	22.59	1.90	0.09	11.81	6.35a	13.69a
2019	21.54	1.78	0.08	12.16	6.27ab	12.73ab
Effect of sampling time^†^	NS	NS	NS	NS	**	***
**SS2**						
2013	19.90c	1.73	0.11	11.47	6.05	9.82c
2014	21.72abc	1.95	0.14	11.12	6.21	11.31bc
2015	20.20bc	1.81	0.15	11.19	6.21	11.29bc
2016	21.96abc	1.93	0.12	11.41	6.2	12.09abc
2017	20.54bc	1.76	0.13	11.67	6.23	12.63ab
2018	22.75ab	1.98	0.14	11.49	6.38	14.06a
2019	23.26a	1.98	0.11	11.72	6.3	13.42ab
Effect of sampling time^†^	**	NS	NS	NS	NS	**
**LP**						
2013	19.94	1.74	0.08	11.46ab	6.08b	10.18c
2014	20.61	1.83	0.08	11.28ab	6.08b	10.67bc
2015	19.1	1.73	0.09	11.05ab	6.18b	10.58bc
2016	21.01	2.06	0.08	10.38b	6.30ab	11.93abc
2017	20.67	1.76	0.08	11.69ab	6.32ab	12.74ab
2018	22.09	1.85	0.06	11.89a	6.47a	13.55a
2019	21.1	1.78	0.06	11.85a	6.31ab	12.32abc
Effect of sampling time^†^	NS	NS	NS	**	**	**
**PL**						
2013	20.14b	1.74	0.13	11.56ab	6.15	10.32b
2014	20.91ab	1.84	0.09	11.34ab	6.45	11.86ab
2015	19.73b	1.77	0.09	11.17b	6.51	11.77ab
2016	20.89ab	1.87	0.08	11.18b	6.65	12.90ab
2017	20.81ab	1.74	0.09	11.92ab	6.47	13.52a
2018	21.73ab	1.84	0.07	11.77ab	6.63	14.14a
2019	23.06a	1.92	0.07	11.98a	6.61	13.95a
Effect of sampling time^†^	*	NS	NS	*	NS	**

*Significant at the 0.05 probability level. **Significant at the 0.01 probability level. ***Significant at the 0.001 probability level. ^†^Values with the same lower case letters in a column and within treatment are not significantly different at P < 0.05. DM, Dry Matter.

### Chemical characteristics of organic fertilizers

Each organic material was dried and ground to 1 mm to measure the pH, organic C, total N, and P. The total N and organic C contents were measured by elemental analysis (NA 1500, Fison Instrument, San Carlos, CA, United States) after additional grinding to 200 μm (Retsch SM 2000, Haan, Germany). The mean characteristics of the organic fertilizers measured between 2014 and 2019 are given in Supplementary Table 1. The organic fertilizers were representative of the most common recycled waste in agriculture, particularly in Reunion Island. They were analyzed before each application. Considering all sampling times, both sewage sludge materials (SS1 and SS2) were characterized by a high dry matter content (91.6 and 79.1% on average, respectively), a high pH (11.8 and 9.4) and a low K concentration (1.7 and 3.9 g kg^–1^ DM). Liquid pig manure (LP) had a low dry matter content (3.7% of DM on average), a low pH (4.1) and a high K concentration (99.7 g kg^–1^ DM). Poultry litter (PL) had a low P concentration (14.6 g kg^–1^ DM on average) and a high organic carbon content (410.2 g kg^–1^ DM).

Total DNA was extracted from 1 g (dry weight) of soil using a single procedure standardized by the GenoSol platform (INRAE, Dijon, France)^3^ (Terrat et al., 2012). A highly positive linear relationship exists between soil DNA recovery and C biomass measurements (Ranjard et al., 2003), so the DNA concentrations of the crude extracts were determined by electrophoresis in 1% agarose gel stained with ethidium bromide using a calf thymus DNA standard curve, and used as estimates of microbial biomass (Dequiedt et al., 2011). After quantification, 100 μL of crude DNA extract were separated from the residual impurities, particularly humic substances, using the purification steps of the Nucleospin Soil kit (Macherey-Nagel GmbH & Co., KG, Düren, Germany). Purified DNA concentrations were finally measured using the Quantifluor (Promega, Lyon, France) staining kit, according to the manufacturer’s instructions.

### High throughput sequencing of 16S rRNA gene sequences

For bacterial and archaeal diversity, a 440-base 16S rRNA fragment was amplified from each DNA sample (5 ng) with the corresponding primers: F479 (5′-CAG CMG CYG CNG TAA NAC-3′) and R888 (5′-CCG YCA ATT CMT TTR AGT-3′) as previously described (Tardy et al., 2014). For each sample, 5 ng of DNA were used for a 25 μL PCR conducted under the following conditions: 94°C for 2 min, 35 cycles of 30 s at 94°C, 52°C for 30 s, and 72°C for 1 min, followed by 7 min at 72°C.

All PCR products were purified using the Agencourt^®^ AMPure^®^ XP kit (Beckman Coulter, Italy, Milano) and quantified with the Quantifluor (Promega, Lyon, France) staining kit according to the manufacturer’s instructions.

A second PCR was performed with the purified PCR products (7.5 ng of DNA for bacteria and archaea), with 10-bp multiplex identifiers added to the 5’ end of the primers for the specific identification of each sample and the prevention of PCR biases. The second PCR conditions were the same than previously described but with only seven cycles. PCR products were purified with the MinElute PCR purification kit (Qiagen NV) and quantified with the Quantifluor (Promega, Lyon, France) staining kit according to the manufacturer’s instructions. Equal amounts of each sample were pooled and then cleaned with the SPRI (Solid Phase Reverse Immobilization Method) using the Agencourt^®^ AMPure^®^ XP kit (Beckman Coulter, Italy, Milano). The pool was finally sequenced with a MiSeq Illumina instrument (Illumina Inc., San Diego, CA) operating with V3 chemistry and producing 250 bp paired-reads (GenoScreen).

### Bioinformatic analysis of 16S rRNA gene sequences

Bioinformatic analyses were performed using BIOCOM-PIPE v.20 (Djemiel et al., 2020). First, all the 16S rRNA raw reads were sorted according to the multiplex identifier sequences. Next, all raw sequences were checked and discarded if (i) they contained an ambiguous base (Ns), (ii) their length was less than 350 nucleotides, and (iii) the exact primer sequences were not found. Then, we applied a rigorous dereplication step (i.e., a clustering of strictly identical sequences). The dereplicated reads were aligned using Infernal tool (Cole et al., 2009), and clustered into operational taxonomic units (OTUs) using a similarity threshold of 95%. The threshold at 95% is more adapted for the targeted amplicon (Djemiel et al., 2020; Terrat et al., 2020). A filtering step was carried out to remove chimeras based on the quality of their taxonomic assignments. Finally, the retained reads were homogenized by random selection (10,000 reads for 16S rRNA gene sequences) to compare the datasets efficiently and avoid biased community comparisons. More detailed information about sequencing data quality is given in Supplementary Figure 1.

The retained high-quality reads were used for taxonomy-independent analyses, to determine alpha-diversity metrics (e.g., richness), and taxonomy-based analysis is performed using USEARCH (Edgar, 2010) against the SILVA 16S rRNA reference database (r132) (Quast et al., 2012). The raw datasets are available in the EBI database system under project accession number PRJEB52689.

### Statistical analyses

The soil physico-chemical characteristics and the fertilization practices, *a.k.a.* explanatory variables potentially involved in the variations of soil microbial indices were first tested for autocorrelation. Explanatory variables with | *r*| > 0.6 were excluded from further analyses, and the following soil variables were retained: organic carbon content, C/N ratio, pH, P, clay, and sand, together with the following fertilization variables: organic carbon inputs, mineral nitrogen inputs, mineral phosphorous inputs, organic potassium inputs, and cumulated organic carbon inputs (Cumul_Corg). These variables were used to evaluate whether the soil characteristics and the fertilization practices significantly discriminated the different treatments over time. For this purpose, a principal component analysis was performed using the *dudi.pca* function of the *ade4* package in R (Chessel et al., 2004). Discrimination between treatments, years or treatments x years was tested using a Monte Carlo permutation test (1,000 permutations, *bca* and *randtest* function, *ade4* package). The significance threshold was set at *P* < 0.001.

Alpha diversity was analyzed using Hill numbers generated by vegan package (Chao et al., 2010; Oksanen et al., 2013). The Hill numbers make it possible to interpret alpha diversity in a linear way by progressively taking into account the abundance of OTUs (*q*-value), between rare and dominant OTUs. Thus, *q* = 0 represents richness, *q* = 1 the exponential of Shannon entropy and *q* = 2 the inverse Simpson index.

The variations of the soil microbial biomass and diversity indices (Hill indices q0, q1, and q2) over time were tested for each organic material treatment by means of a Kruskal-Wallis test with Bonferroni correction. The significance threshold was set at *P* < 0.05. For each soil microbial index exhibiting significant differences over time within an organic material treatment, a variance partitioning approach was used to decipher which soil physico-chemical characteristics and fertilization practices were involved. For this purpose, the most parsimonious additive model was selected from null to full model according to a forward selection procedure by minimizing the Akaike information criterion (*rda* and *ordistep* functions, *vegan* package). Once the model was selected, the variance inflation factor was checked (*vif.cca* function, *vegan* package) (Oksanen et al., 2013). Variables with vif > 4 were excluded, and the selection procedure was run a second time.

The composition of the bacterial and archaeal communities associated with each treatment and sampling time were compared using Non-Metric multiDimensional Scaling approach (NMDS). The relative abundances of bacterial and archaeal phyla were determined, and then a Bray-Curtis dissimilarity matrix was computed. Then, NMDS was applied using the *metaMDS* function in the *vegan* package (Oksanen et al., 2013; 1,000 permutations). Individuals were plotted in the NMDS space according to NMDS1 and NMDS2 axes, which depicted the differences in the bacterial and archaeal community compositions. The significance of the differences between treatments and sampling times were assessed using a PERMANOVA approach was used to test for the relative effect of organic material treatment, time and organic material treatment x time (*adonis2* function, *vegan* package; 1,000 permutations, significance threshold: *P* < 0.001). To better understand the sources of variations of the composition of bacterial and archaeal communities, phylum, soil physico-chemical properties and fertilization practices data were fitted in the NMDS space using the *envfit* function (*vegan* package; 1,000 permutations). Only the variables with *P* < 0.001 were retained and plotted in the NMDS space. To better understand these variations, a linear discriminant analysis effect size (LEfSe) was used to detect the taxa with significant differential abundances among the soil fertilization types for all years (Segata et al., 2011). Briefly, the LEfSe method is based on a first step using the non-parametric Kruskal-Wallis rank sum test to identify the statistically different bacterial groups, followed by a linear discriminant analysis step (LDA scores > 3) to evaluate the effect size of each differentially abundant taxon. The cladogram represented the taxa with different abundance among the different groups. To better characterize these biomarkers, major phyla and genera were selected with LDA scores higher than 4 and their relative abundance was used to evaluate the responses of the bacterial and archaeal community to organic-inorganic inputs over time.

All statistical analyses were carried out with R studio (RStudio, Version 1.4.1717, RStudio Inc., Boston, Massachusetts, United States) using R software (R version 4.1.1).

## Results

### Soil physico-chemical characteristics

**FIGURE 1:**
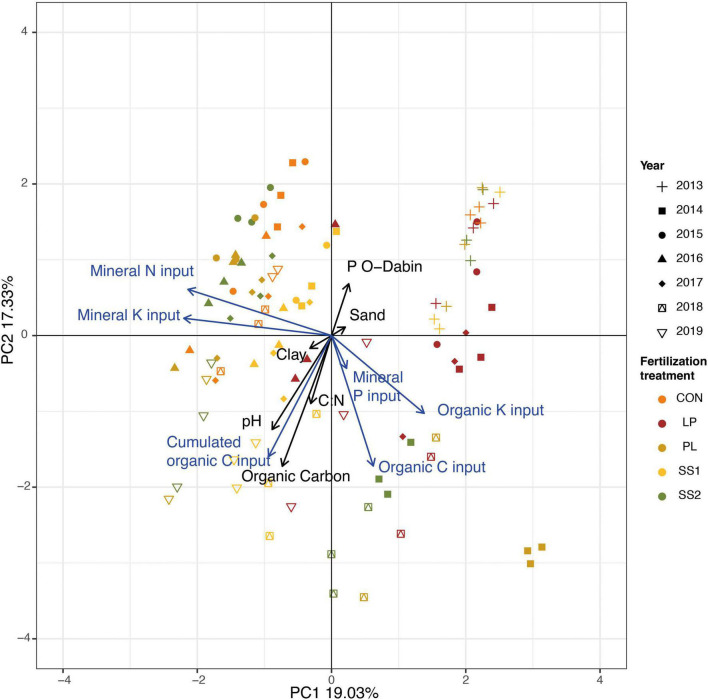
Principal component analysis of the soil physico-chemical characteristics and fertilization practices in the experimental site of La Mare (Reunion Island) fertilized solely with inorganic fertilizer (control, CON), or fertilized with four different organic materials (SS1 and SS2, sewage sludge inputs; LP, liquid pig manure; PL, poultry litter). Discrimination between treatments, years or treatments x years was tested using a Monte Carlo permutation test (1,000 permutations). The black arrows are the soil physico-chemical characteristics, and the blue are the fertilizers physico-chemical characteristics.

The Principal Component Analysis (PCA) of the soil physico-chemical characteristics and fertilization practices highlighted that the soil samples were discriminated according to treatments and years (Figure 1; Monte Carlo permutation test: *P* < 0.001). Discrimination between treatments was mainly explained by fertilization practices: organic inputs (C and K) vs. mineral input (N), whereas inter-annual variations were mainly explained by the soil physico-chemical characteristics and the cumulated organic C inputs (Figure 1). More precisely, as described in Table 1, the soil organic carbon significantly increased in SS2 and PL plots (by 15% on average), 5 and 6 years after the start of the experiment, respectively (*P* < 0.05, Table 1). The total N content remained stable over time, irrespective of the treatment (*P* > 0.05; Table 1). The soil pH increased significantly (by 6% on average) only in SS1 and LP plots, 5 years after the start of the experiment (*P* < 0.05, Table 1). CEC significantly increased in all organically fertilized plots (by 37% on average) 3–4 years after the start of the experiment (*P* < 0.01; Table 1).

### Effects of organic fertilization on the microbial community

**FIGURE 2:**
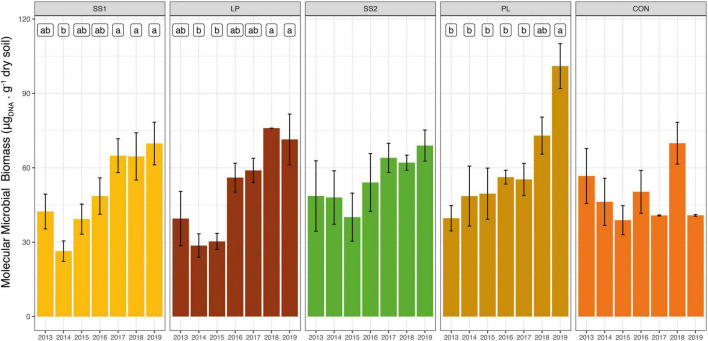
Dynamic of molecular microbial biomass in soil from La Mare (Reunion Island) fertilized or not (inorganic fertilizer control, CON) with four different organic materials (SS1 and SS2, sewage sludge inputs; LP, liquid pig manure; PL, poultry litter). Different letters indicate significant differences based on Kruskal-Wallis test with Bonferroni correction (*P* < 0.05).

The estimated soil molecular biomass did not change over time in the control and SS2 plots (*P* > 0.05, Figure 2), but significantly increased in the other organically fertilized plots (SS1, LP, and PL) (by 35–45% on average) 3, 4, and 5 years after the first fertilizer application, respectively (*P* < 0.01; Figure 2). In the SS1 and LP treatments, soil microbial biomass variations were mainly related to fertilization practices. Cumul_Corg represented 55 and 59% of the explained variance of the soil microbial biomass in the SS1 and LP plots, respectively (Table 2). Soil parameters were also important: the sand and soil carbon contents represented 23 and 41% of the explained variance in the SS1 and PL plots, respectively (Table 2).

**TABLE 2:** Effects of environmental conditions on the soil molecular microbial biomass (BMM) and the bacterial—archaeal diversity indices (Hill number with an order 1 and 2) of soils from La Mare (Reunion Island) according to the fertilizer input (CON, inorganic fertilized control; SS1 and SS2, sewage sludge inputs; LP, liquid pig manure; PL, poultry litter).

Parameters	Treatment	Explanatory variables	Standardized regression coefficient	Variance (%)	Model variance (%)
BMM	SS1	Cumul_Corg	0.185	54.9	86.8
		Sand	−0.128	23.4	
		Min_N_inputs	−0.145	8.5	
		Residual	−	13.2	
	LP	Cumul_Corg	0.375	59.1	59.1
		Residual	−	40.9	
	SS2	−	−	0	0
	PL	C_soil	0.175	41	41
		Residual	−	59	
	CON	−	−	0	0
Hill_q1	SS1	Cumul_Corg	0.188	44.3	44.3
		Residual	−	55.7	
	LP	−	−	0	0
	SS2	C_soil	0.154	43.7	43.7
		Residual	−	56.3	
	PL	Cumul_Corg	0.270	35.8	74.4
		CN	0.315	19	
		Sand	0.083	9.2	
		Interactions	−	10.4	
		Residual	−	25.6	
	CON	C_soil	0.316	44.9	58
		Sand	0.138	13.1	
		Residual	−	41.9	
Hill_q2	SS1	pH	0.297	38.6	38.6
		Residual	−	61.4	
	LP	−	−	0	0
	SS2	−	−	0	0
	PL	−	−	0	0
	CON	C_soil	0.286	37.7	37.7
		Residual		62.3	

**FIGURE 3:**
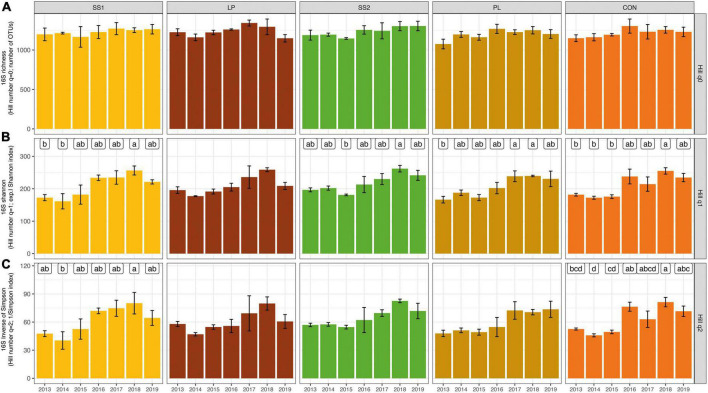
Dynamic of the bacterial and archaeal community based on Hill numbers [Hill number with an order 0 **(A)**, 1 **(B)**, and 2 **(C)**] in soil from La Mare (Reunion Island) fertilized or not (CON, inorganic fertilizer control) with four different organic materials (SS1 and SS2, sewage sludge inputs; LP, liquid pig manure; PL, poultry litter). For each treatment, different letters indicate significant differences between years based on Kruskal-Wallis test with Bonferroni correction (*P* < 0.05).

The dynamic of the bacterial and archaeal diversity indices throughout the experiment is showed in Figure 3. Irrespective of the treatment, the soil bacterial and archaeal Hill number with an order 0 (*q* = 0, *aka* H_0_) diversity index did not change over time (*P* > 0.05, Figure 3). The Hill number with an order 1 (*q* = 1, *aka* H_1_) and 2 (*q* = 2, *aka* H_2_) indices in general increased in the SS1 and control plots 4 years after the first fertilizer application (*P* < 0.001; Figure 3). In plots SS1, H_1_ index variations were mainly related to fertilization practices. Cumul_Corg represented 44% of the explained variance of the H_1_ index variations in plots SS1 (Table 2). In the control plots, H_1_ index variations were mainly related to soil parameters: soil carbon content and sand represented 45 and 13% of the explained variance, respectively (Table 2). For the H_2_ index, variations were exclusively related to soil parameters: soil pH and soil carbon content represented 39 and 38% of the explained variance in the SS1 and control plots, respectively (Table 2).

**FIGURE 4:**
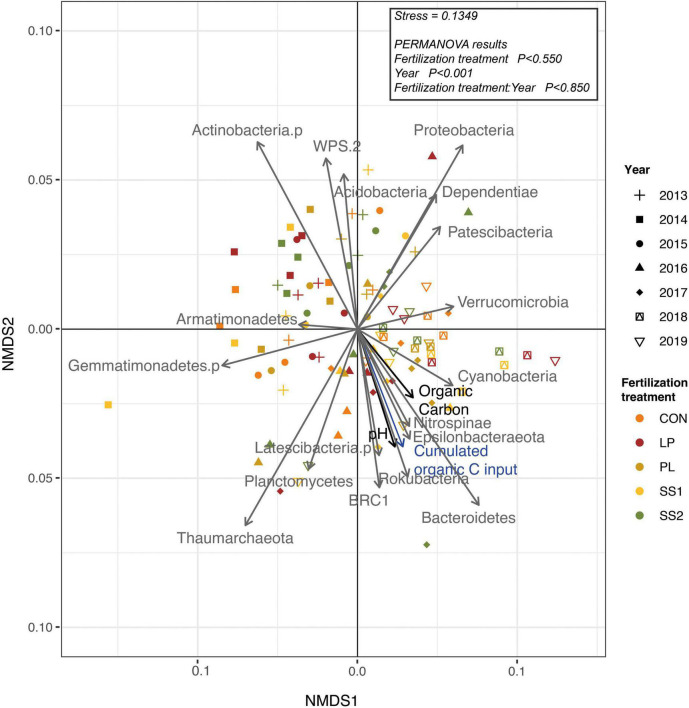
Non-metric Multi-Dimensional Scaling (NMDS) ordination plot derived from Bray Curtis dissimilarity distances for the bacterial and archaeal community in soil from La Mare (Reunion Island) fertilized or not (CON, inorganic fertilizer control) with four different organic materials (SS1 and SS2, sewage sludge inputs; LP, liquid pig manure; PL, poultry litter).

**FIGURE 5:**
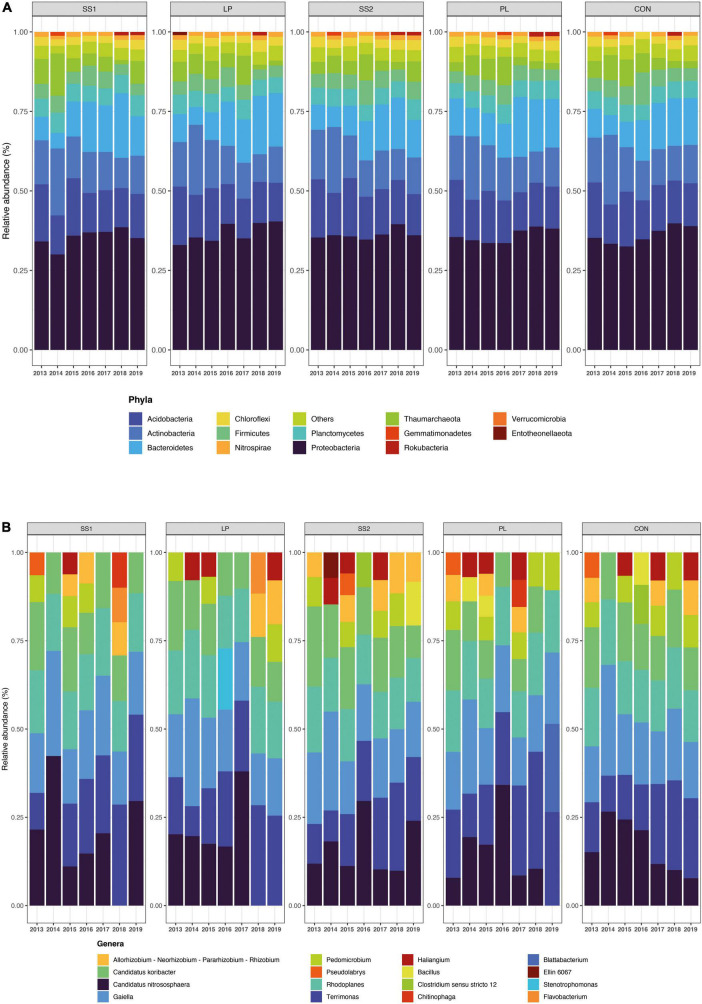
Relative abundance of archaea and bacteria at the phylum **(A)** and genus **(B)** level across years for the different fertilization treatments (CON, inorganic fertilizer control) with four different organic materials (SS1 and SS2, sewage sludge inputs; LP, liquid pig manure; PL, poultry litter). Relative taxonomic abundances below 1 or 2% were grouped in “Others” for the phylum and genus levels, respectively. Unknown taxa were excluded from genus plotting.

**FIGURE 6:**
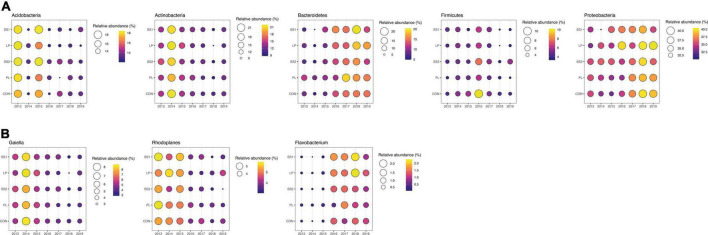
Bubble plots showing the relative abundance of the major bacterial phyla **(A)** and genera **(B)** significantly different depending on fertilization practices (CON, inorganic fertilizer control; SS1 and SS2, sewage sludge inputs; LP, liquid pig manure; PL, poultry litter) over time after a LEfSe analysis. Presented phyla and genera were selected based on LEfSe analysis allowing to detect the taxa with significant differential abundances among the soil fertilization types for all years.

The NMDS ordination highlighted that the structure of the bacterial and archaeal community was more influenced by time (*P* < 0.001; PERMANOVA; Table 3 and Figure 4) than by the fertilization type (*P* > 0.05; PERMANOVA, Table 3 and Figure 4). Thus, repeated fertilizer applications over time tended to modify the abundance of the bacterial and archaeal phyla and genera (Figures 4, 5). The LEfSe analysis allowed identifying major bacterial and archaeal phyla and genera presenting the strongest response to fertilization practices over time (Figure 6). Five phyla were significantly impacted: *Acidobacteria, Actinobacteria, Bacteroidetes, Firmicutes*, and *Proteobacteria* (Figure 6 and Supplementary Figures 2–6). In general, 1 year after the start of the experiment (2014), the relative abundance of most bacterial and archaeal phyla shifted whatever the fertilization type, with a return to the initial state 1 year later (2015). At the phylum level, this shift was characterized by a decrease of *Acidobacteria* and *Bacteroidetes* and an increase of *Actinobacteria* and *Firmicutes* (Figures 5, 6). Whatever the treatment, *Proteobacteria* was a major phylum with high relative abundance over time. This phyla recorded, however, smallest variations over time compared to other phyla (Figures 5, 6). In 2016, a second shift was observed. From this period, *Bacteroidetes* and *Proteobacteria* increase lastingly in all plots, whereas a decrease of *Acidobacteria* and *Actinobacteria* was observed (Figures 5, 6). Three major bacterial genera presented the strongest response to fertilization practices over time, following various patterns. *Gaiella* (*Actinobacteria*) increased 1 year after the start of the experiment, and then decreased gradually over time and in all fertilization types (Figure 6). *Rhodoplanes* (*Proteobacteria*) were steadily found highly abundant from 2013 to 2015 whatever the fertilization type, and then declined slightly until the end of the study (Figure 6). Finally, *Flavobacterium* (*Bacteroidetes*) was mostly absent whatever the fertilization type during the first 3 years and then increased significantly, in particular the SS1 and LP plots (Figure 6).

**TABLE 3:** PERMANOVA analysis results to depicted the differences in the bacterial and archaeal community compositions of soils from La Mare (Reunion Island) according to the organic material treatment, time, and organic material treatment × time (1,000 permutations, significance threshold: *P* < 0.001).

	*Df*	SS	*R* ^2^	*F*	*P*-value
Fertilization treatment	4	0.144	0.0312	0.964	0.542
Year	6	1.265	0.274	5.657	0.001
Fertilization treatment × year	24	0.817	0.177	0.913	0.845
Residual	64	2.385	0.517	-	-
Total	98	4.611	1	-	-

Df, Degrees of freedom; SS, Sum of Squares; R2, Determination coefficient; F, Fisher’s F-value; P-value, P-value of Fisher’s F.

## Discussion

Repeated use of organic and inorganic fertilizers over several years improves crop productivity. To better understand the impact of these fertilization practices on soil fertility, we assessed the dynamic of the soil bacterial and archaeal communities in response to different repeated mixed organic-inorganic fertilizer inputs in a field experiment. To our knowledge, this has never been assessed before.

Repeated applications of mixed organic and inorganic materials induced lasting modifications of the soil chemical properties under some fertilization conditions, with increases of the soil organic carbon content and the pH. Along with the control plots that only received inorganic fertilization, the SS2 and PL plots received the greatest quantity of inorganic inputs. As mentioned by Giacometti et al. (2013), the increase of the soil organic carbon content under mid-term mineral fertilizer application may be an indirect effect of higher plant biomass production and C return to soils. However, the soil organic carbon content did not increase in the control plots. This suggests that the increase of the soil organic carbon content was not only due to the sole indirect effect of mineral fertilization. In parallel, several studies performed following several years of repeated fertilizer inputs have shown that the combination of organic and inorganic inputs increases the soil organic carbon content compared with inorganic fertilization (Hao et al., 2008; Zhong et al., 2010; Chakraborty et al., 2011; Li et al., 2015, 2017; Sun et al., 2015; Francioli et al., 2016; Kumar et al., 2017; Guo et al., 2019; Diallo et al., 2021; Feder, 2021a). Therefore, we suppose that the increased soil organic carbon content observed in SS2 and PL resulted from mixed organic-inorganic fertilizer inputs. The quality of organic material applied could also explain the lasting increase of the soil organic carbon content for these two treatments. The C:N ratio is good proxy to assess the quality of organic materials applied (labile or recalcitrant) (Vallejo, 1993). Thus, SS2 and PL could have more recalcitrant organic carbon (C:N = 7.43 and 9.68, respectively, Supplementary Table 1) which could explain increase of soil organic carbon contain.

Five and 6 years after the start of the experiment (2018), a significant increase of the pH value was observed only in the SS1 and LP, respectively. Plots SS1 and LP were the only two plots that received organic fertilization yearly. Our findings are consistent with previous studies highlighting that repeated applications of mixed organic and inorganic materials increase the soil pH (Zhong et al., 2010; Dong et al., 2014; Hartmann et al., 2015; Zhao et al., 2016; Wang et al., 2017).

As observed in previous studies, our results highlight that some repeated mixed organic-inorganic fertilizer inputs (SS1, LP and PL) induce a lasting increase of the soil microbial biomass (Hao et al., 2008; Gu et al., 2009; Chakraborty et al., 2011; Li et al., 2015; Francioli et al., 2016; Kumar et al., 2017; Guo et al., 2019) occurring 4–6 years after the start of the experiment. Compared to a low frequency of organic material application (every 3 years; SS2 and PL), a yearly application (SS1 and LP) lastingly increased the soil microbial biomass. Interestingly, a positive correlation between the soil carbon content and the soil microbial biomass was observed in the PL plots (Table 2). PL was applied every 3 years, and had a high C:N ratio (9.68 on average). This suggests that poultry litter would be more resistant to decomposition and would have a higher C sequestration potential. Thus, low-frequency application of an organic material with a high C:N ratio (e.g., PL) also led to a lasting increase of the soil carbon content that allowed for optimal growth of soil microbes, and in turn an increase of the soil microbial biomass.

The bacterial and archaeal richness index (i.e., H_0_) of the organically inorganically fertilized plots and control plots that only received inorganic fertilization did not evolve over time. Literature results are not congruent about the effect of fertilization practices on soil alpha-diversity. Some works report a higher bacterial richness in soil fertilized with a mixed organic-inorganic fertilization input compared with a soil solely fertilized with inorganic material (Hartmann et al., 2015; Sun et al., 2015), whereas others show opposite results (Gu et al., 2009). The quantities of organic and inorganic materials were similar across all studies. However, as pointed out by Hartmann et al. (2015), these different results could be due to the use of different methods (primer sequences, bioinformatics analysis), the metric itself, and most importantly the experimental design. Regarding H_1_ and H_2_ indices, both increased significantly in SS1 and control plots 4 years after the first fertilizer input; and H_1_ similarly tended to increase in SS2 and PL plots. Altogether, this suggested that these fertilization practices stimulate abundant OTUs more than LP treatment, and that control and SS1 treatments stimulated dominant OTUs. Except for H_2_ in SS1 plot, variations of H_1_ or H_2_ were mainly related either to soil organic carbon content or cumulated carbon inputs. The increase of H_1_ and H_2_ indices may be related to organic fertilizer application with high C:N ratio in SS1, SS2, and PL plots but also by returning straw to soil in all plots. This would suggest that increasing soil carbon resources may increase some abundant and dominant phyla as supported by Francioli et al. (2016); leading to an increase in H_1_ and H_2_. In SS1 plots, changes in H_2_ were not related to carbon resources but to soil pH variations. This may be explained a confounding effect of soil pH and organic fertilizer annual inputs or cumulated organic carbon inputs since slight but not significant correlations between these variables were observed.

As observed by Durrer et al. (2017), the dominant bacterial and archaeal phyla in tropical soils under sugarcane cultivation were *Proteobacteria, Acidobacteria, Actinobacteria, Bacteroidetes, Planctomycetes, Firmicutes, Thaumarchaeota*, and *Chloroflexi*. In the present study, the structure of the bacterial and archaeal community evolved over time whatever fertilizer input: *Acidobacteria*, and *Actinobacteria* decreased, while *Bacteroidetes* and *Proteobacteria* increased. A strong modification of bacterial and archaeal community composition occurred in 2014 but this returned to its initial state in 2015. This suggests that the experimental set up impacted strongly the community composition likely because of soil plowing (Lienhard et al., 2014). On the other hand, since 2016, bacterial and archaeal community composition was lastingly modified regarding to 2015 whatever the fertilization practice, with slight variations between 2016 and 2019. During this period, there were no major physical perturbations but sugarcane straw were systematically reallocated. This supplemental organic material input may have reduced the relative effect of each organic treatments on bacterial and archaeal community composition. This is not in agreement with other studies reporting significant differences between the composition of soil bacterial communities associated to different fertilizer inputs (Hartmann et al., 2015; Francioli et al., 2016; Wang et al., 2017; van der Bom et al., 2018). Indeed, these authors highlight that organic fertilization stimulates copiotrophic phyla such as *Firmicutes* and *Proteobacteria*, known to prefer nutrient-rich environments and involved in the degradation of complex organic compounds. In contrast, the soils that did not receive manure harbored distinct microbial communities enriched in oligotrophic organisms adapted to nutrient-limited environments, such as *Acidobacteria* and *Bacteroidetes*. *Actinobacteria* have been described abundant both in organically (Poulsen et al., 2013; Riber et al., 2014) and inorganically fertilized soils (Francioli et al., 2016). This phylum is often described as having a copiotrophic lifestyle (Fierer et al., 2007; Ramirez et al., 2012). At the end point of our study (2019), our results are not in accordance with these previous works that as altogether, our results show no differences in bacterial community composition between treatments (Figure 4, PERMANOVA, *P* < 0.0850), despite several years of fertilizer application. The similar soil bacterial and archaeal community dynamics of inorganic and mixed organic-inorganic fertilizer inputs found in the present study are surprising compared to the literature. A stimulation of oligotrophic phyla such as *Acidobacteria* and *Bacteroidetes* in the control plots was expected. However, as sugarcane residues were returned to the soil, they also contributed to organic fertilization in the plots that only received inorganic fertilization. This could explain the similar dynamic of bacterial and archaeal community structures in plots receiving different fertilizer inputs.

The lasting modification over time of the bacterial and archaeal community composition was also observed at the genus level. Three genera were implied: *Gaiella* (*Actinobacteria*), *Rhodoplanes* (*Proteobacteria*), and *Flavobacterium* (*Bacteroidetes*). Except for *Flavobacterium*, the relative abundance of all of these genera decreased over time. Little information is available in the literature on these genera. However, *Gaiella* and *Flavobacterium* would have a higher relative abundance in soil fertilized with inorganic material (van der Bom et al., 2018; Cai et al., 2020), whereas *Rhodoplanes* would tend to increase in soil fertilized with organic material. The difference with our results could be explained by the mixed fertilization practices in our trial: although all plots received inorganic fertilization, all of them also received organic material inputs through sugarcane residue restitution, which added to organic fertilizer when applied.

The present study indicates that the bacterial and archaeal community of a tropical soil evolves over time, with few changes in the first years following the start of the experiment, and a lasting modification 3–6 years after it (depending on the soil microbial indicator considered). The structure of the bacterial and archaeal community was more influenced by time than by the nature of organic input or the fertilization practice (frequency and applied quantity).

## Data availability statement

The datasets presented in this study can be found in online repositories. The names of the repository/repositories and accession number(s) can be found below: https://www.ebi.ac.uk/ena/browser/view/PRJEB52689.

## Author contributions

SS-B and FF planned the study and performed the lab work. FF coordinated the sampling. SS-B, FF, CD, and NC analyzed the data and provided general guidance. SS-B wrote the manuscript. FF, CD, and NC contributed to reviewing the manuscript. All authors contributed to the article and approved the submitted version.

## Footnotes

1
https://www6.inrae.fr/valor-pro/Les-sites-experimentaux/SOERE-PRO-Reunion

2
https://www6.hautsdefrance.inrae.fr/las

3
https://www2.dijon.inrae.fr/plateforme_genosol/
