# Supplementary figures and images for: Dynamic of bacterial and archaeal diversity in a tropical soil over 6 years of repeated organic and inorganic fertilization
Source: Front Microbiol. 2022 Aug 16;13:943314. doi: 10.3389/fmicb.2022.943314 (PMC9425033; doi:10.3389/fmicb.2022.943314)

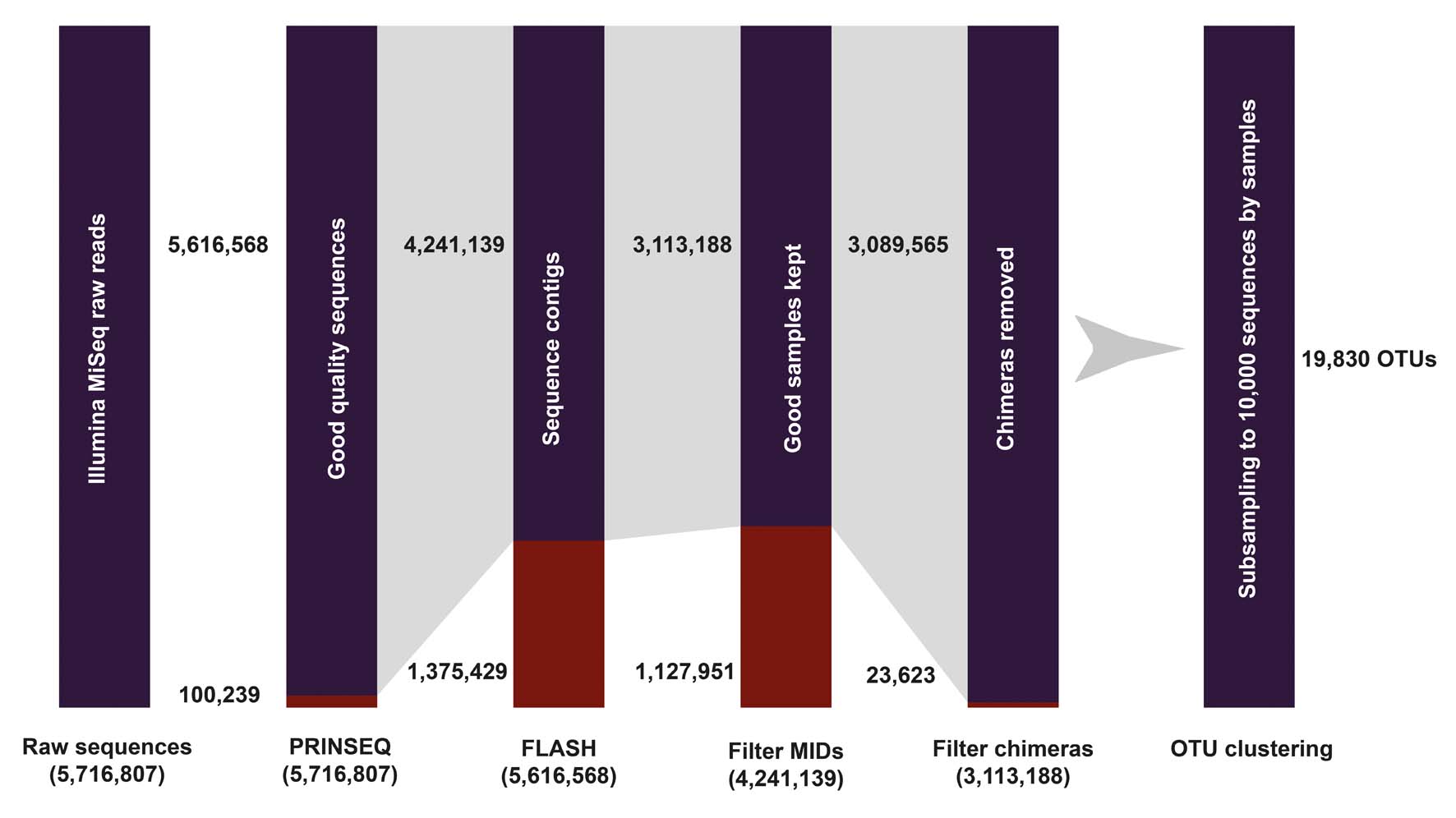

Supplement: Supplementary file 1 [file Image_1.JPEG]

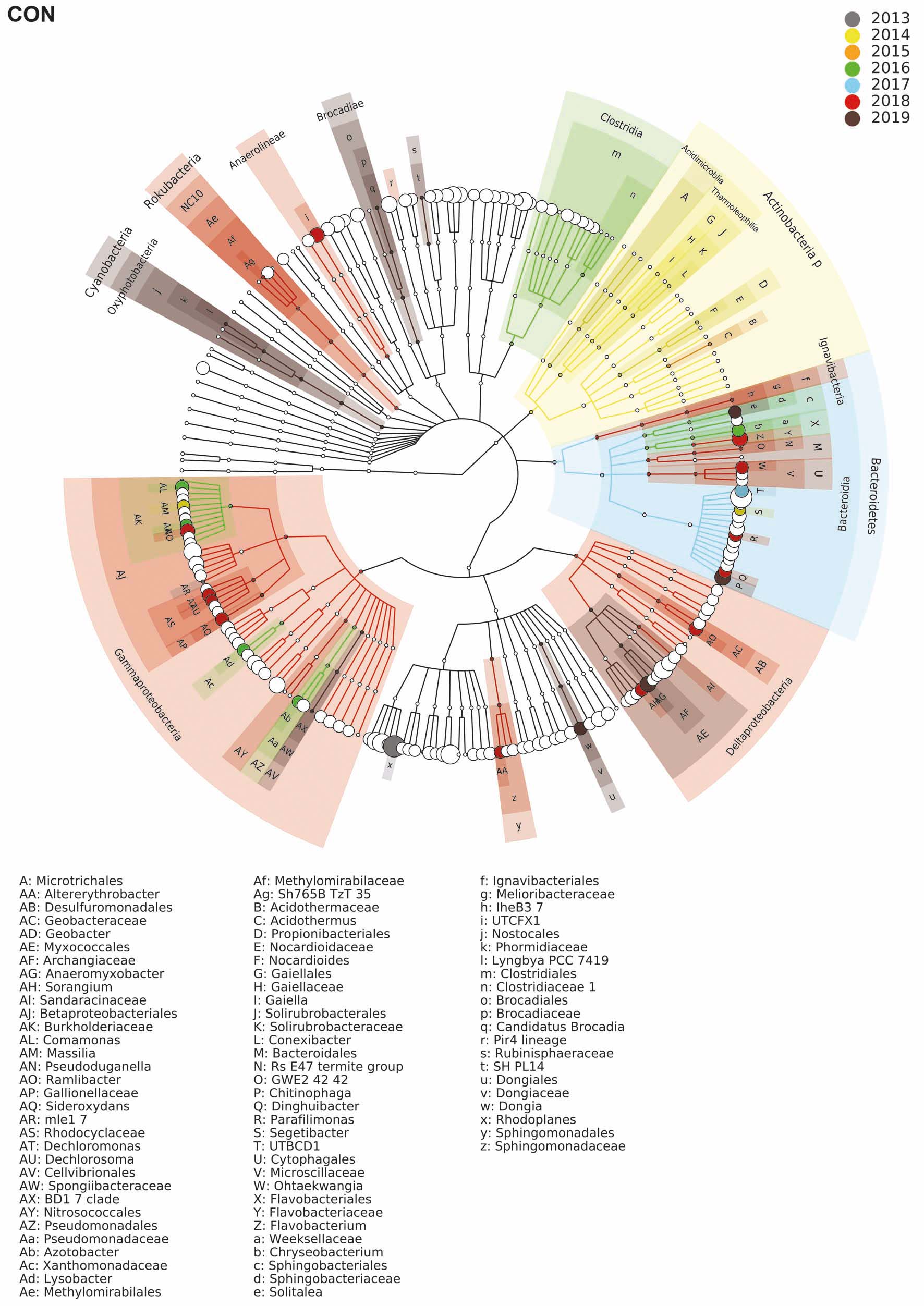

Supplement: Supplementary file 2 [file Image_2.JPEG]

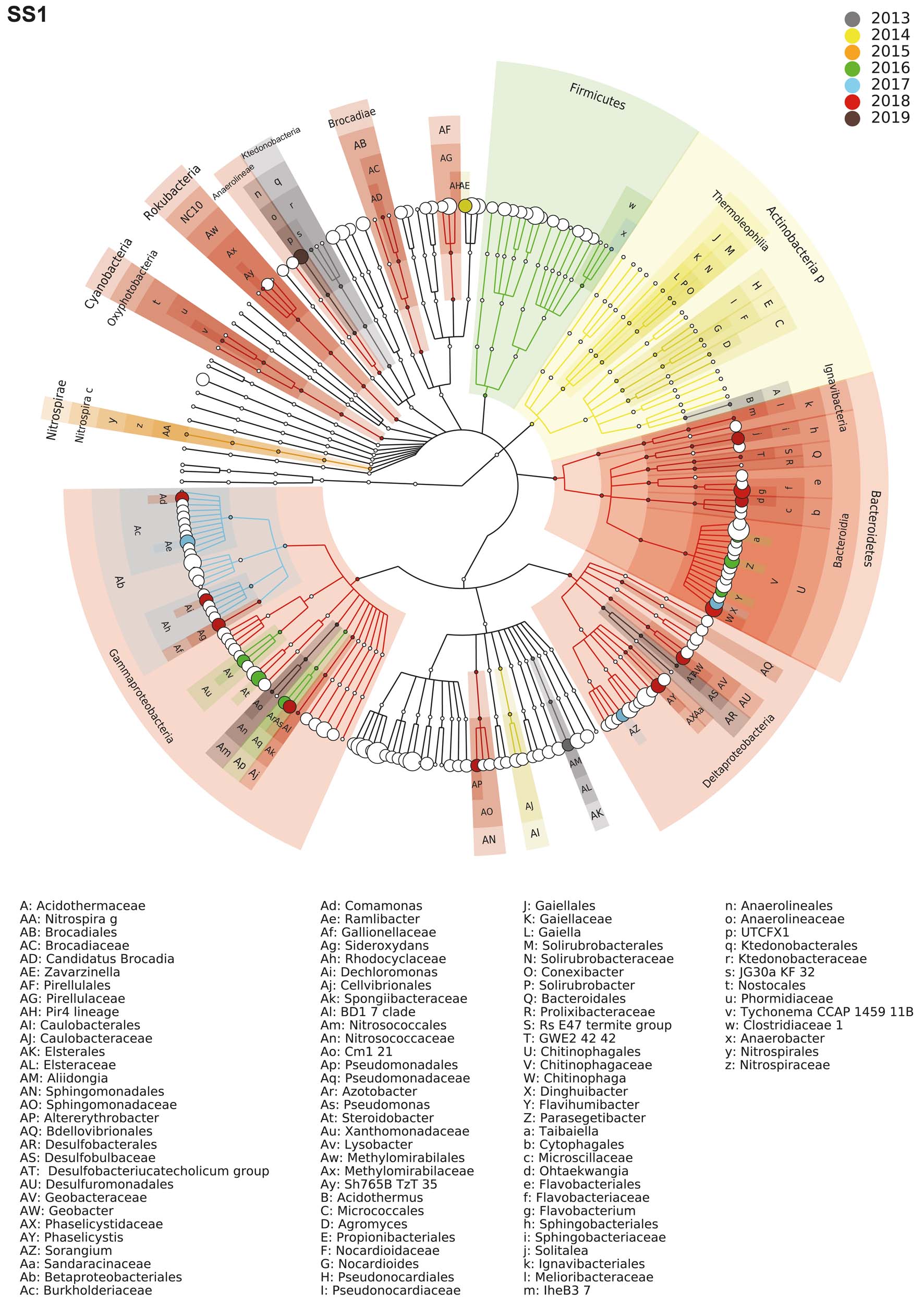

Supplement: Supplementary file 3 [file Image_3.JPEG]

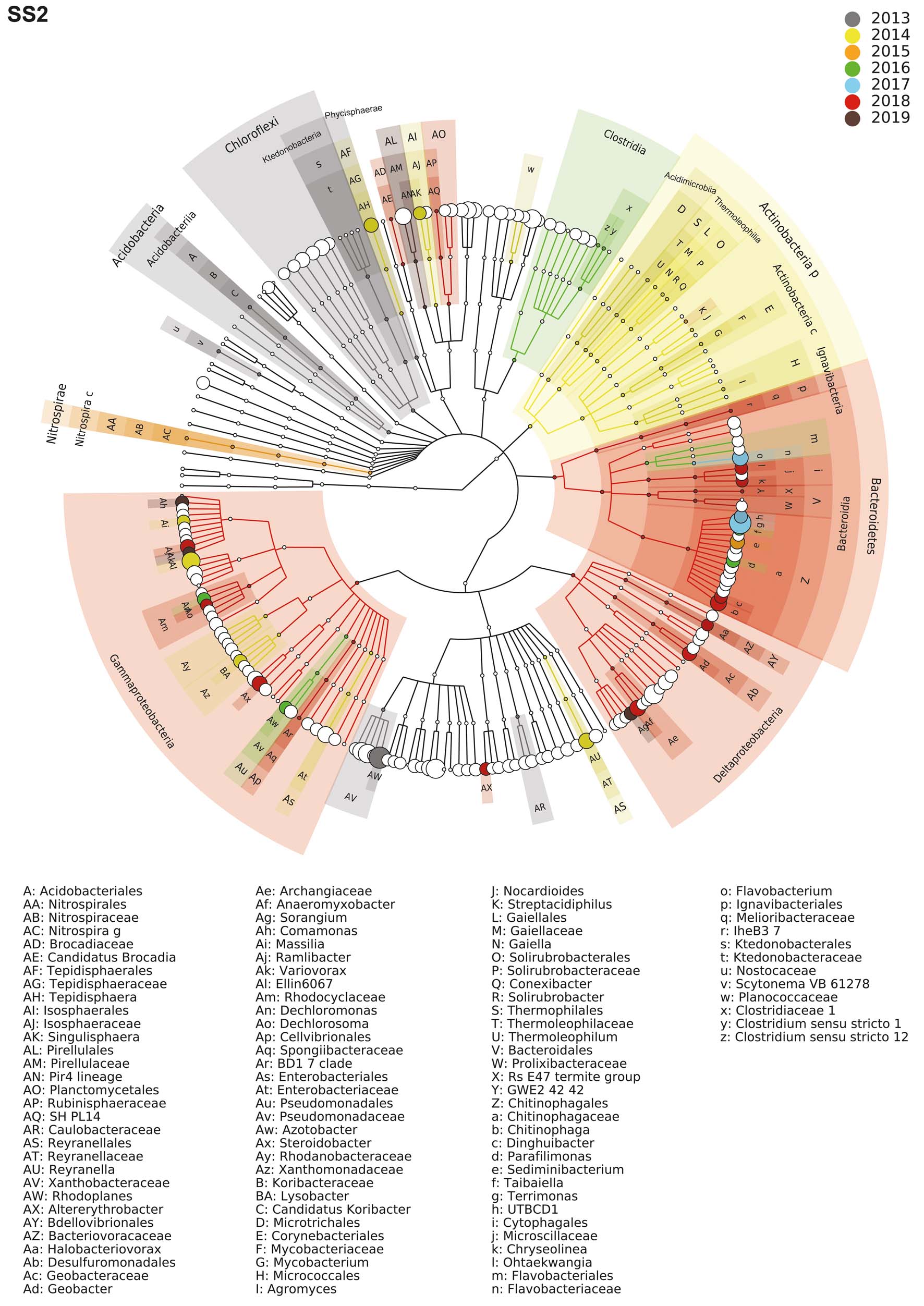

Supplement: Supplementary file 4 [file Image_4.JPEG]

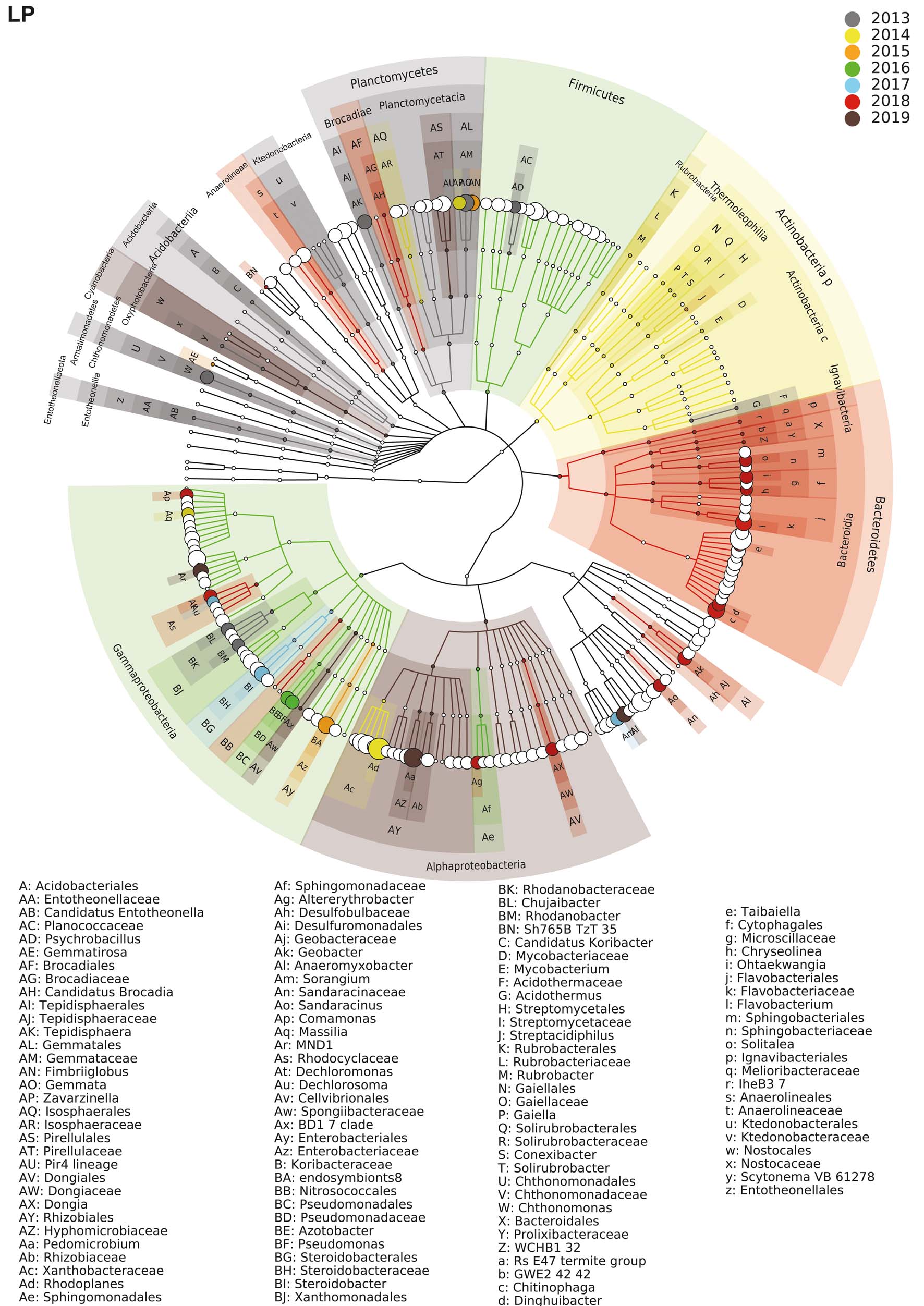

Supplement: Supplementary file 5 [file Image_5.JPEG]

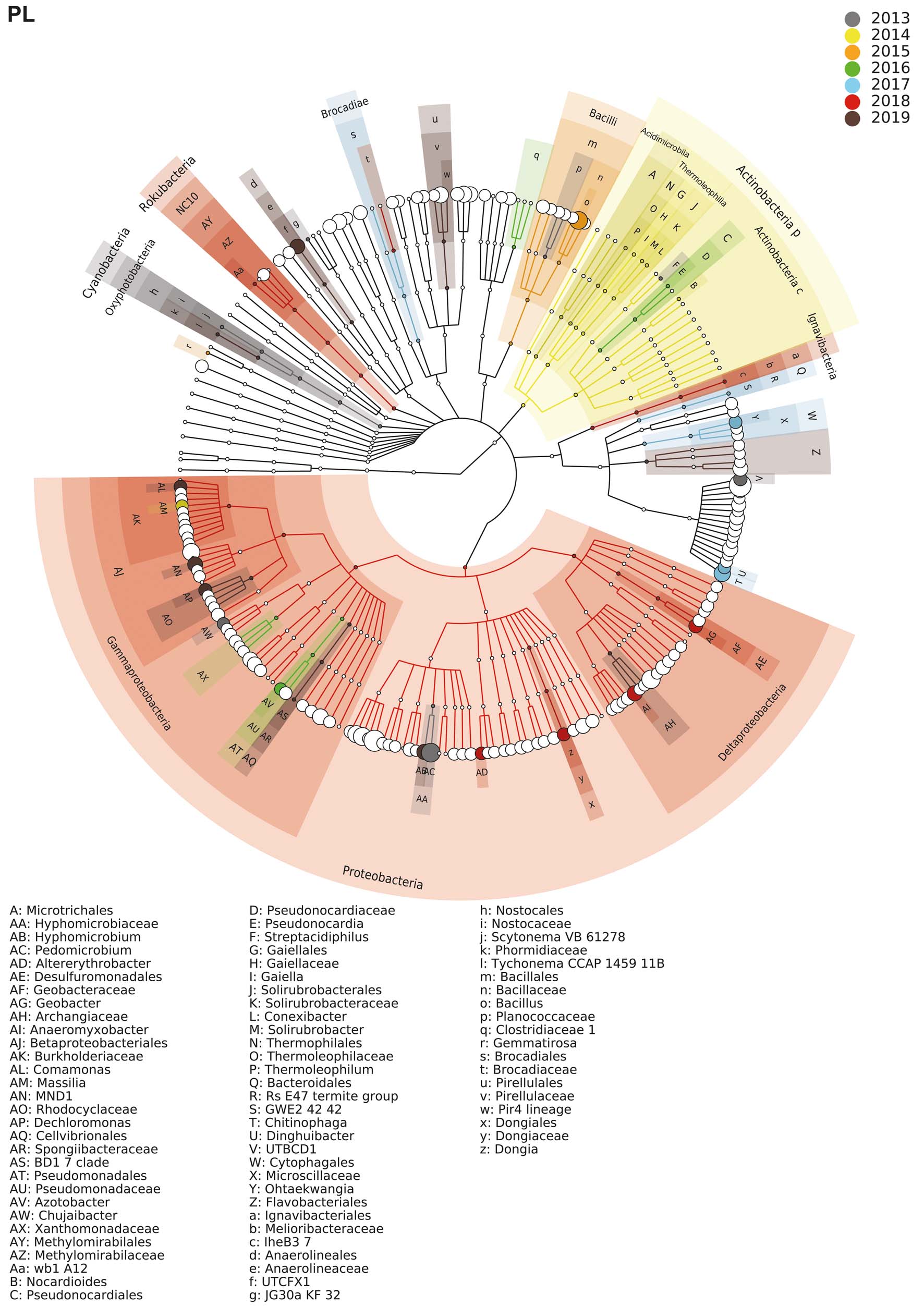

Supplement: Supplementary file 6 [file Image_6.JPEG]
