# Supplementary material for: Dynamic of bacterial and archaeal diversity in a tropical soil over 6 years of repeated organic and inorganic fertilization
Source: Front Microbiol. 2022 Aug 16;13:943314. doi: 10.3389/fmicb.2022.943314 (PMC9425033; doi:10.3389/fmicb.2022.943314)
Supplement: Supplementary file 7 [file Data_Sheet_1.docx]

**Dynamic of bacterial and archaeal diversity in a tropical soil over 6 years of repeated organic and inorganic fertilization**

**S. Sadet-Bourgeteau^1^*, C. Djemiel^1^, N. Chemidlin Prévost-Bouré^1^ and F. Feder^2,3^**

^1^UMR 1347 Agroecologie, Institut Agro Dijon, INRAE, Université de Bourgogne, Université de Bourgogne-Franche Comté, F-21000 Dijon, France

^2^CIRAD, UPR Recyclage et Risque, F-34398 Montpellier, France

^3^Recyclage et risque, Univ Montpellier, CIRAD, Montpellier, France

*** Correspondence:**Sophie Sadet-Bourgeteau
[sophie.bourgeteau-sadet@agrosupdijon.fr](mailto:sophie.bourgeteau-sadet@agrosupdijon.fr)

# Supplementary Table 1. Organic and inorganic fertilizers applied in soil from La Mare (Reunion Island) between 2014 and 2019 (CON, inorganic fertilizer control; SS1 and SS2, sewage sludge inputs; LP, liquid pig manure; PL, poultry litter).

| Plots | Organic fertilization | | | | | | |  | Mineral fertilization | | | |
| --- | --- | --- | --- | --- | --- | --- | --- | --- | --- | --- | --- | --- |
|  | Date of application | Applied quantity | C | N | C:N | P | K |  | Date of application | N | P | K |
|  |  | t ha^-1^ FM | kg ha^-1^ DM | | | |  |  |  | kg ha^-1^ | |  |
| CON | Feb 2014 | - | - | - | - | - | - |  | Feb 2014 | 168.00 | 58.00 | 233.30 |
|  | Nov 2014 | - | - | - | - | - | - |  | Nov 2014 | 168 | 58.08 | 233.33 |
|  | Nov 2015 | - | - | - | - | - | - |  | Nov 2015 | 168 | 46.72 | 233.33 |
|  | Nov 2016 | - | - | - | - | - | - |  | Dec 2016 | 145.00 | 36.68 | 200.00 |
|  | Oct 2017 | - | - | - | - | - | - |  | Oct 2017 | 144.81 | 36.68 | 200.00 |
|  | Oct 2018 | - | - | - | - | - | - |  | Oct 2018 | 144.00 | 36.68 | 200.00 |
|  | Oct 2019 | - | - | - | - | - | - |  | Dec 2019 | 144.00 | 36.68 | 200.00 |
|  | **Total** | **-** | **-** | **-** | **-** | **-** | **-** |  | **Total** | **1081.81** | **309.52** | **1499.96** |
| SS1 | Feb 2014 | 3.7 | 1020.85 | 117.9 | 8.66 | 92.18 | 5.14 |  | Feb 2014 | 104.85 | 0.00 | 227.54 |
|  | Nov 2014 | 3.5 | 1045.49 | 137.1 | 7.63 | 81.63 | 3.06 |  | Dec 2014 | 106.30 | 9.09 | 227.59 |
|  | Nov 2015 | 2.95 | 891.7 | 130.84 | 6.82 | 71 | 4.18 |  | Dec 2015 | 109.65 | 0.00 | 229.21 |
|  | Nov 2016 | 2.32 | 700.38 | 108.42 | 6.46 | 58.3 | 2.99 |  | Dec 2016 | 93.92 | 0.00 | 194.81 |
|  | Oct 2017 | 2.43 | 2246.89 | 325.37 | 6.91 | 195.51 | 15.21 |  | Dec 2017 | 95.63 | 0.00 | 193.74 |
|  | Oct 2018 | 2.44 | 749.81 | 109.87 | 6.82 | 63.23 | 5.1 |  | Dec 2018 | 94.92 | 0.07 | 195.93 |
|  | Oct 2019 | 2.38 | 736.51 | 110.97 | 6.64 | 63.85 | 4.36 |  | Dec 2019 | 96.25 | 0.02 | 195.82 |
|  | **Total** | **17.29** | **7391.63** | **1040.5** | **7.1** | **625.7** | **40.04** |  | **Total** | **701.52** | **9.176** | **1464.64** |
| SS2 | Feb 2014 | 9.9 | 2893.81 | 351.88 | 8.22 | 257.57 | 15.49 |  | Feb 2014 | 0.00 | 82.92 | 217.84 |
|  | Nov 2014 | - | - | - |  | - | - |  | Dec 2014 | 168.00 | 0.00 | 233.33 |
|  | Nov 2015 | - | - | - |  | - | - |  | Dec 2015 | 168.00 | 0.00 | 233.33 |
|  | Nov 2016 | - | - | - |  | - | - |  | Dec 2016 | 144.00 | 0.00 | 200 |
|  | Oct 2017 | 14.88 | 2691.29 | 399.58 | 6.74 | 238.15 | 61.49 |  | Dec 2017 | 56.57 | 0.00 | 132.12 |
|  | Oct 2018 | - | - | - |  | - | - |  | Dec 2018 | 144.00 | 0.00 | 200.00 |
|  | Oct 2019 | - | - | - |  | - | - |  | Dec 2019 | 144.00 | 0.00 | 200.00 |
|  | **Total** | **24.78** | **5585.1** | **751.46** | **7.43** | **495.72** | **76.98** |  | **Total** | **824.57** | **82.92** | **1416.62** |
| LP | Feb 2014 | 95 | 706.65 | 173.61 | 4.07 | 63.09 | 175.79 |  | Feb 2014 | 83.07 | 14.48 | 0.00 |
|  | Nov 2014 | 56.7 | 172.97 | 111.47 | 1.55 | 12.24 | 145.43 |  | Dec 2014 | 79.03 | 0.00 | 0.00 |
|  | Nov 2015 | 28 | 677.13 | 114.3 | 5.92 | 56.04 | 59.48 |  | Dec 2015 | 121.72 | 0.00 | 177.06 |
|  | Nov 2016 | 91 | 210.74 | 169.48 | 1.24 | 114.46 | 250.99 |  | Dec 2016 | 55.00 | 0.00 | 129.16 |
|  | Oct 2017 | 76.7 | 1295.66 | 253.97 | 5.1 | 101.16 | 155.45 |  | Dec 2017 | 80.38 | 0.00 | 0.00 |
|  | Oct 2018 | 58.5 | 422.95 | 132.41 | 3.19 | 31.32 | 109.34 |  | Dec 2018 | 84.61 | 0.00 | 140.82 |
|  | Oct 2019 | 102 | 270.41 | 179.13 | 1.51 | 19 | 207.73 |  | Dec 2019 | 58.39 | 0.00 | 76.95 |
|  | **Total** | **507.9** | **3756.51** | **1134.4** | **3.31** | **397.31** | **1104.2** |  | **Total** | **562.2** | **14.48** | **523.99** |
| PL | Feb 2014 | 10.4 | 2743.18 | 273.7 | 10 | 92.03 | 221.84 |  | Feb 2014 | 10.61 | 171.54 | 0.00 |
|  | Nov 2014 | - | - | - |  | - | - |  | Dec 2014 | 168.00 | 0.00 | 233.33 |
|  | Nov 2015 | - | - | - |  | - | - |  | Dec 2015 | 168.00 | 0.00 | 233.33 |
|  | Nov 2016 | - | - | - |  | - | - |  | Dec 2016 | 144.00 | 0.00 | 200.00 |
|  | Oct 2017 | 7.39 | 577.62 | 69.32 | 8.33 | 22.41 | 59.1 |  | Dec 2017 | 14.93 | 69.24 | 0.00 |
|  | Oct 2018 | - | - | - |  | - | - |  | Dec 2018 | 144.00 | 0.00 | 200.00 |
|  | Oct 2019 | - | - | - |  | - | - |  | Dec 2019 | 144.00 | 0.00 | 200.00 |
|  | **Total** | **17.79** | **3320.8** | **343.02** | **9.68** | **114.44** | **280.94** |  | **Total** | **793.54** | **240.78** | **1066.66** |

FM: fresh Matter; DM: Dry Matter

**Supplementary Table 2.** Sugarcane yields (T/ha) in La Mare (Reunion Island) between 2014 and 2019, for each treatment (CON, inorganic fertilizer control; SS1 and SS2, sewage sludge inputs; LP, liquid pig manure; PL, poultry litter), for each years; standard deviation are in brackets.

|  | 2014 | 2015 | 2016 | 2017 | 2018 | 2019 |
| --- | --- | --- | --- | --- | --- | --- |
| SS1 | 65 (16) | 104 (22) | 107 (7) | 118 (11) | 107 (13) | 106 (7) |
| SS2 | 50 (25) | 116 (14) | 128 (12) | 105 (15) | 104 (2) | 100 (10) |
| LP | 57 (17) | 104 (17) | 107 (17) | 104 (16) | 97 (23) | 85 (10) |
| PL | 59 (16) | 99 (12) | 128 (5) | 102 (17) | 90 (8) | 90 (5) |
| CON | 51 (15) | 98 (5) | 106 (17) | 98 (14) | 88 (20) | 79 (10) |

**Supplementary Table 3.** List of pesticide treatments conducted in La Mare (Reunion Island) between 2013 and 2019, on all treatments (CON, inorganic fertilizer control; SS1 and SS2, sewage sludge inputs; LP, liquid pig manure; PL, poultry litter / 1.3 hectares).

| 1/9/2013 | In 400 L: 1 L of 2,4-dichlorophenoxyacetic acid (46%) + 3 L of pendimethalin (38.7%) + 2.5 L of (20 g/L benoxacor + 400 g/L S-metolachlore + 40 g/L mesotrione) |
| --- | --- |
| 3/17/2013 | In 400 L: 1 L of 2,4-dichlorophenoxyacetic acid (46 %) |
| 12/16/2013 | In 400 L: 4 L of glyphosate (42%) + 1 L of 2,4-dichlorophenoxyacetic acid (46%) |
| 1/5/2014 | In 400 L: 0.5 L of metribuzin (52.2%) + 350 mL of mesotrione (40%) + 1 L of (20 g/L benoxacor + 400 g/L S-metolachlore + 40 g/L mesotrione) |
| 1/7/2014 | In 320 L: 2.9 L of glyphosate (42%) |
| 3/5/2014 | In 400 L: 20 g of isoxaflutole (75%) + 6 L of pendimethalin (38.7%) + 1 L of 2,4-dichlorophenoxyacetic acid (46%) |
| 1/27/2015 | In 400 L: 1 L of 2,4-dichlorophenoxyacetic acid (46%) + 700 mL of mesotrione (40%) + 550 g of metribuzin (52.2%) |
| 2/6/2015 | In 100 L: 170 mL of 2,4-dichlorophenoxyacetic acid (46%) + 170 mL of mesotrione (40%) + 185 g of metribuzin (52.2%) |
| 2/5/2016 | In 300 L: 1.5 L of 2,4-dichlorophenoxyacetic acid (46%) + 1.5 L of mesotrione (40%) + 0.5 L of fluroxypyr-meptyl (288.2 g/L) |
| 12/15/2016 | In 400 L: 2 L of pendimethalin (38.7%) + 300 g of metribuzin (52.2%) |
| 2/2/2017 | In 400 L: 1 L of 2,4-dichlorophenoxyacetic acid (46%) + 700 mL of mesotrione (40%) + 550 g of metribuzin (52.2%) |
| 2/14/2017 | In 160 L: 1.44 L of glyphosate (42%) |
| 6/8/2017 | In 400 L: 1 L of 2,4-dichlorophenoxyacetic acid (46%) |
| 1/20/2018 | In 200 L: 25 mL of 2,4-dichlorophenoxyacetic acid (46%) + 16 g of metribuzin (52.2%) + 60 mL of (20 g/L benoxacor + 400 g/L S-metolachlore + 40 g/L mesotrione) |
| 5/5/2018 | In 400 L: 1 L of 2,4-dichlorophenoxyacetic acid (46%) + 620 g of metribuzin (52.2%) + 2.5 L of (20 g/L benoxacor + 400 g/L S-metolachlore + 40 g/L mesotrione) |
| 11/14/2018 | In 400 L: 1 L of 2,4-dichlorophenoxyacetic acid (46%) + 620 g of metribuzin (52.2%) + 2.5 L of (20 g/L benoxacor + 400 g/L S-metolachlore + 40 g/L mesotrione) |
| 3/2/2019 | In 400 L: 1 L of 2,4-dichlorophenoxyacetic acid (46%) + 620 g of metribuzin (52.2%) + 2.5 L of (20 g/L benoxacor + 400 g/L S-metolachlore + 40 g/L mesotrione) |
| 6/5/2019 | In 400 L: 750 mL of mesotrione (40%) + 1 L of 2,4-dichlorophenoxyacetic acid (46%) + 625 g of metribuzin (52.2%) |

**Supplementary Figure. 1.** The number of sequences kept and removed at each processing step. To normalize our samples we subsampled at 10,000 reads per sample.

**Supplementary Figure. 2.** Taxonomic tree from LefSe analysis for archaeal and bacterial communities over time for control plots (CON). Colors identify the years and point size represent the relative abundance of the group. Codes are reported in the legend and refer to different taxonomic levels from phylum to genus.

**Supplementary Figure. 3.** Taxonomic tree from LefSe analysis for archaeal and bacterial communities over time for sewage sludge materials plots (SS1). Colors identify the years and point size represent the relative abundance of the group. Codes are reported in the legend and refer to different taxonomic levels from phylum to genus.

**Supplementary Figure. 4.** Taxonomic tree from LefSe analysis for archaeal and bacterial communities over time for sewage sludge materials plots (SS2). Colors identify the years and point size represent the relative abundance of the group. Codes are reported in the legend and refer to different taxonomic levels from phylum to genus.

**Supplementary Figure. 5.** Taxonomic tree from LefSe analysis for archaeal and bacterial communities over time for liquid pig manure plots (LP). Colors identify the years and point size represent the relative abundance of the group. Codes are reported in the legend and refer to different taxonomic levels from phylum to genus.

**Supplementary Figure. 6.** Taxonomic tree from LefSe analysis for archaeal and bacterial communities over time for poultry litter plots (PL). Colors identify the years and point size represent the relative abundance of the group. Codes are reported in the legend and refer to different taxonomic levels from phylum to genus.
